# Association of Brain Iron Overload With Brain Edema and Brain Atrophy After Intracerebral Hemorrhage

**DOI:** 10.3389/fneur.2020.602413

**Published:** 2020-12-18

**Authors:** Ran Liu, Haoran Zhang, Shuangjuan Cheng, Yuyao Sun, Haijiao Li, Jiangxi Xiao, Yining Huang

**Affiliations:** ^1^Department of Neurology, Peking University First Hospital, Beijing, China; ^2^Department of Radiology, Peking University First Hospital, Beijing, China

**Keywords:** cerebral hemorrhage, magnetic resonance imaging, iron overload, susceptibility weighted image (SWI), brain edema

## Abstract

**Objective:** This study evaluated iron overload after intracerebral hemorrhage (ICH) using ESWAN sequences.

**Methods:** This single-center prospective observational cohort study enrolled supratentorial ICH patients. MRI was obtained with a 3.0-T scanner at day 1, day 14, day 30, and follow-up (300 days or later). R2* mapping was generated based on the ESWAN. R2* value of the ipsilateral side represented iron deposition, and the R2* value of the contralateral side served as control. R2* value was adjusted by volume and used to assess total iron overload. Brain edema was measured on T2 FLAIR-weighted images. Brain atrophy was calculated as the contralateral hemisphere volume minus the injured hemisphere volume.

**Results:** Twnety-seven patients with a spontaneous supratentorial ICH were included in this analysis. The ipsilateral R2* value was 40.27 ± 11.62, 41.92 ± 13.56, and 60.89 ± 14.09 at days 1, 14, and 30, respectively. The R2* value was significantly higher in the ICH side than the contralateral side (*p* < 0.01). Increased R2* value was seen on day 30 compared to day 14 (*p* < 0.01). The R2* value showed logistic decay with the distance to the hematoma margin (*p* < 0.01). Brain edema at day 14 and brain atrophy at follow-up correlated with R2* value adjusted by volume at day 14 (*p* < 0.01).

**Conclusions:** After ICH, the iron deposition in the perihematomal region was progressively increased during the first month. R2* value adjusted by volume predicted acute brain edema and chronic brain atrophy.

## Introduction

Intracerebral hemorrhage (ICH) is a devastating disease with high morbidity and mortality (1). The rupture of a large number of erythrocytes in a hematoma leads to the mass accumulation of hemoglobin, which causes the accumulation of iron in the hematoma and surrounding tissue and subsequently results in iron overload (2). Iron overload provokes a neurotoxic effect and is causally related to edema formation after ICH and delayed neuronal injury (3, 4). In clinical studies, the level of ferritin on admission is associated with poor outcome in patients with ICH (5–7). In animal models, iron chelator has been shown to reduce ICH-induced brain edema, neuronal death, brain atrophy, and neurological deficits (8, 9). A randomized controlled trial revealed that iron chelator is safe but the efficacy of this needs further study (10, 11). To interpret the effects of such interventions, a full understanding of the natural history and clinical significance of iron overload is needed.

A hematoma contains deoxyhemoglobin and hemosiderin, from which the susceptibility effects cause signal decay, resulting in a hypointense signal on T2*-weighted magnetic resonance imaging (MRI) (12). Susceptibility weighted imaging (SWI) has been shown to have a higher sensitivity for detecting iron than conventional T2*-weighted MRI. Furthermore, R2*, the reciprocal of T2*, is near-linear rise with iron concentration, and R2* mapping could estimate perihematomal brain iron content in the rat ICH model (13). However, there remains a lack of studies that detect iron overload in ICH patients using MRI.

Therefore, we aimed to quantify the iron overload in the perihematomal area using SWI during the first 30 days after intracerebral hemorrhage, and investigate the relationship between iron concentration with acute brain edema and chronic brain atrophy.

## Methods

### Subjects

The design of the study has been previously published (14). This study was a single-center prospective observational cohort study enrolling ICH patients. The study was approved by the ethics committee of Peking University Health Science Center and Michigan University Joint Institute, and informed consent was obtained from the patients or their surrogates.

### Inclusion and Exclusion Criteria

The study included consecutive men and non-pregnant women >18 years old with a primary supratentorial hematoma, admitted within 24 h after symptom onset. The detailed exclusion criteria have been previously published (14).

### Clinical Assessments

Demographic data, vascular risk factors, and clinical characteristics were prospectively collected. The modified Boston criteria were used to assess possible/probable cerebral amyloid angiopathy (CAA) on MRI scans. If cortical superficial siderosis and/or multiple cortical microbleeds, or a single lobar hemorrhage, were present, patients were classified as “probable CAA” (15, 16). At their follow-up visit, cognitive function was evaluated with the Mini-mental State Examination (MMSE) and Montreal Cognitive Assessment (MoCA).

### Imaging Protocol

All patients underwent non-contrast head computed tomography (CT) on admission. MRI was performed on a GE Discovery MR750 3.0-T scanner at day 1 (24 ± 12 h), day 14 (14 ± 3 days), day 30 (3–6 weeks), and follow-up (300 days or later). All ESWAN sequences were acquired using a 3D-enhanced T2* susceptibility-weighted angiography contrast flow compensated multiecho gradient echo sequence, which was performed with the following parameters: repetition time (TR) 22.7 ms, echo time (TE) 3.2/5.6/7.9/10.3/12.6/15.0/17.3/19.7 ms, and slice thickness 2 mm. The parameters of routine MR imaging were as follows: T1 weighted images (T1WI): TR 1750 ms, TE 25 ms; T2 weighted images (T2WI): TR 5,092 ms, TE 92.5 ms; T2 Fluid Attenuated Inversion Recovery (T2-FLAIR): TR 8,400 ms, TE 150 ms; and T2* weighted images (T2*WI): TR 300 ms, TE 7.7 ms. These routine sequences were acquired with 5-mm-thick contiguous sections.

### Image Analysis

**Figure 1:**
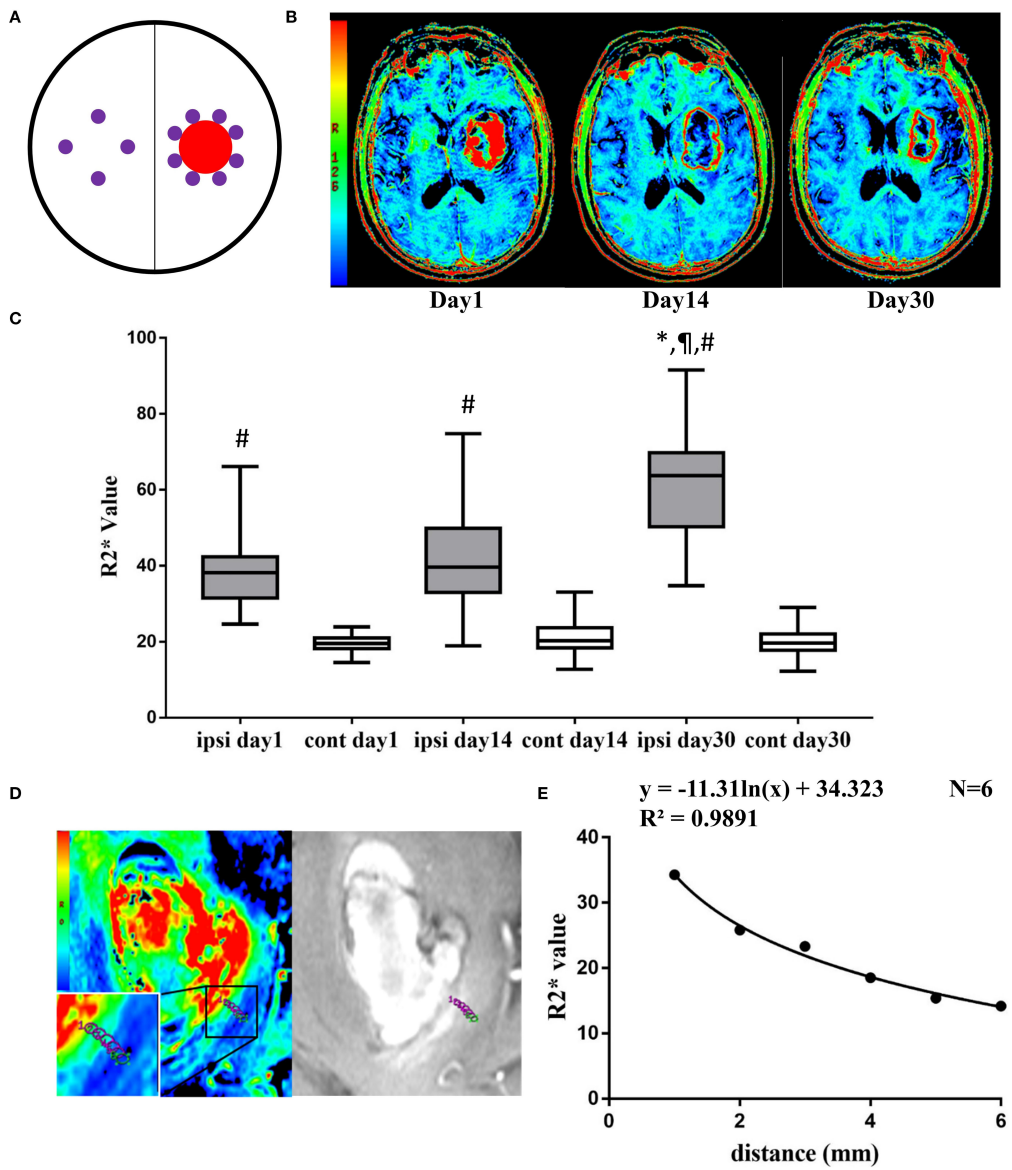
**(A)** Diagram of ipsilateral and contralateral ROIs (purple circle regions) after ICH (red circle region). **(B)** Representative pictures of pseudocolor R2* images at day 1, day 14, and day 30 after ICH. **(C)** Ipsilateral and contralateral R2* value at day 1, day 14, and day 30. Values are shown as box-plot. ^#^*p* < 0.01 vs. contralateral side at same time point. ^*,¶^*p* < 0.01 vs. ipsilateral side at day 1 and day 14. *N* = 17 at day 1, *N* = 22 at day 14, *N* = 15 at day 30. **(D)** Representative pictures of pseudocolor R2* images and T2*WI at day 14, 6 ROIs were drawn from adjacent to the distal part of the hematoma. **(E)** The logistic regression of R2* value and the distance to hematoma edge.

The initial hematoma volume was measured based on the admission CT using the ABC/2 method. Using postprocessor software (ESWAN software, GE), magnitude images and high-pass filtered phase images were automatically presented, which were transformed to pseudocolor images and achieved R2* images. The R2* region of interests (ROIs) with an area of 1 mm^2^ were acquired on the section with the largest hematoma. Edge of hematoma was defined on SWI image. Ipsilateral R2* ROIs were drawn along the edge of the hematoma on R2* images, and the mirrored area in the contralateral hemisphere served as the intraindividual control (Figure 1A). The number of ROIs varied according to the size of the hematoma. For the hemorrhagic side, 9.35 ± 1.37 ROIs were made on day 1, 7.82 ± 2.04 ROIs were made on day 14, and 8.33 ± 1.80 ROIs were made on day 30. 4 ROIs were made on the contralateral side at each time point. Each ipsilateral and contralateral R2* value was measured and then averaged to obtain a final value. All R2* Values were measured by two neurologists (Dr. Ran Liu and Dr. Haoran Zhang), the interrater correlation coefficients were 0.910 (95% CI, 0.844–0.948) for ipsilateral R2* value and 0.947 (95% CI, 0.911–0.969) for contralateral R2* value.

To investigate the distribution of R2* value in the perihematomal area, ROIs were set perpendicular to the edge of the hematoma. Six ROIs were placed adjunctive to the hematoma continuously from adjacently to distally, and the R2* value of each was measured (Figure 1D).

**Figure 2:**
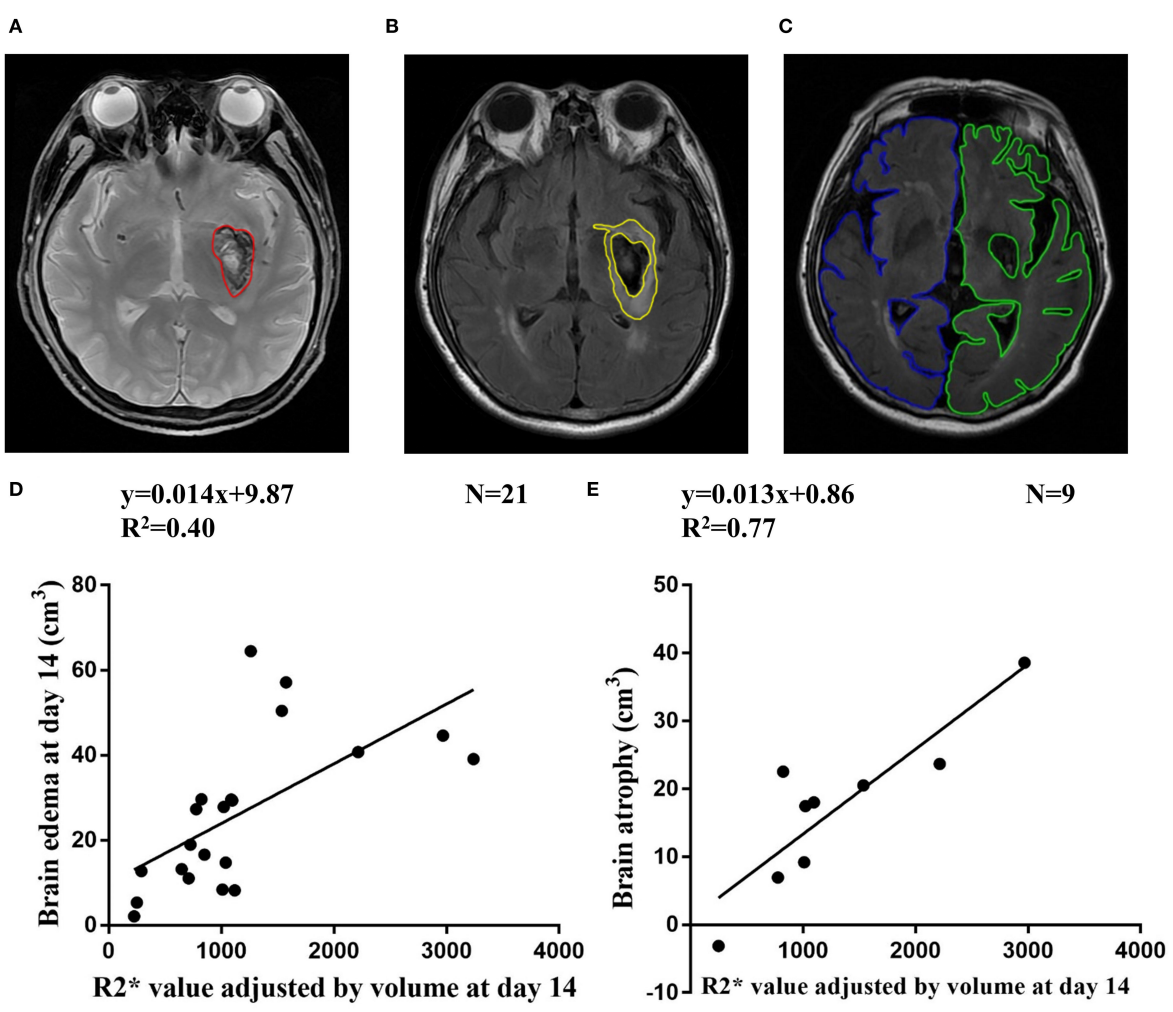
**(A)** Representative ROI of hematoma on T2* weighted images. **(B)** Representative ROI of brain edema on T2 FLAIR-weighted images. **(C)** Representative ROI of non-injured hemisphere volume (blue line) and injured hemisphere (green line) on T2 FLAIR-weighted images at follow-up. **(D)** Correlation between R2* value adjusted by volume at day 14 and brain edema at day 14. **(E)** Correlation between R2* value adjusted by volume at day 14 and brain atrophy at follow-up.

T2*WI and T2 FLAIR images were analyzed with MRIcro version 1.40. Hematoma were outlined on T2*WI images on day 1, day 14, and day 30 (Figure 2A). Brain edema was drawn on T2 FLAIR-weighted images as total lesions minus hematoma lesions (Figure 2B). Hemisphere volume was drawn on T2 FLAIR-weighted images excluding areas of ventricles and damage at follow-up (17). Brain atrophy volume was calculated as the non-injured hemisphere volume minus the injured hemisphere volume (Figure 2C).

We assumed that the hematoma was a sphere with radium (R), surface (S), and volume (V). Then we defined R2* value adjusted by volume = ipsilateral R2* value × S = 4.84 × V2/3 × ipsilateral R2* value.

### Statistical Analysis

All statistical analyses were performed using the Statistical Program for Social Sciences (SPSS) statistical software (version 24.0). Categorical variables were shown as numbers and percentages. Continuous variables were expressed as the mean ± SD or median values and interquartile range as appropriate.

A paired *t*-test was applied to investigate the differences of R2* value between ipsilateral and contralateral sides. The R2* value of the ipsilateral side at different time points were compared with a paired *t*-test. A logistic regression model was used to test for correlations with distance and the corresponding R2* value. Relationships between the R2* value adjusted by volume and brain edema/brain atrophy were assessed with Spearman's correlation. According to the median of R2* value adjusted by volume, patients were divided into two groups. Chi-square test, non-parametric test, or *t*-test were used to compare characteristics between groups. A value of *P* < 0.05 was considered significant.

## Results

### Patient Characteristics

A total of 30 patients with ICH were enrolled between January and November 2014. Three patients were excluded due to poor image quality. The demographic, clinical, laboratory, and radiological characteristics are shown in Table 1. None of the enrolled patients were on anticoagulating therapy before index ICH. All patients underwent a diagnostic CT scan on day 1. Blood pressure was continuously monitored and carefully managed. The systolic blood pressures were <160 mmHg when the first MRI was taken and 140 mmHg when the rest of the MRI were taken. In total, 17, 22, and 15 patients underwent MRI on day 1, day 14 and day 30, respectively. Fifteen patients underwent follow-up MRI, but three patients were excluded because of bilateral ICH, history of cerebral infarction, or new stroke after enrollment.

**Table 1:** Demographic and clinical data for all patients (*N* = 27).

**Demographic data**	
Age, y	59.59 ± 12.19
Male, No. (%)	23 (85.20%)
**Medical history, No. (%)**	
Hypertension	19 (70.40%)
Diabetes	5 (18.50%)
Intracerebral hemorrhage	2 (7.40%)
Smoking	14 (51.90%)
**Etiology of ICH, No. (%)**	
Hypertension	17 (62.96%)
Cerebral amyloid angiopathy	2 (7.40%)
Unknown	8 (29.62%)
**Admission clinical data**	
Glasgow Coma Scale score	15.00 (1.00)
NIHSS score	7.00 ± 4.01
Systolic blood pressure, mm Hg	159.78 ± 26.95
Diastolic blood pressure, mm Hg	98.63 ± 16.12
**Imaging data**	
Lobar location of ICH, No. (%)	5 (18.50%)
Deep location of ICH, No. (%)	22 (81.50%)
Hematoma volume on admission CT, cm^3^	14.46 ± 7.83
Brain edema volume at day 1, cm^3^	17.69 ± 9.65
Brain edema volume at day 14, cm^3^	26.25 ± 17.71
Brain edema volume at day 30, cm^3^	9.76 ± 10.61
Brain atrophy volume at follow-up, cm^3^	13.43 ± 12.49
**Laboratory data**	
Leukocyte, 10^9^/L	6.93 ± 1.71
Hemoglobin, g/L	148.00 (22.00)
Platelets, 10^12^/L	196.30 ± 42.55
Glucose, mmol/L	6.15 (1.47)
TCHO, mmol/L	4.64 (0.77)
TG, mmol/L	1.29 (0.94)
LDL, mmol/L	2.80 ± 0.79
HDL, mmol/L	1.03 (0.36)
Creatinine, μmol/L	90.59 ± 13.31
PT, s	10.30 (1.15)
INR	1.00 (0.09)
Homocysteine, mmol/L	18.95 (17.26)
**Treatment, No. (%)**	
Mannitol	24 (88.89%)
Glycerol fructose	6 (22.22%)
Intravenous antihypertensive drugs	2 (7.40%)
Oral antihypertensive drugs	18 (66.67%)
**3-month outcome**	
Mortality, No. (%)	0 (0%)
Modified rankin score	2.00 (1.00)

### MRI Quantification of Iron Overload in ICH Patients

On day 1, the highest R2* region remained inside the hematoma. At day 14, the highest R2* region was located mainly in the perihematomal area, showing a red ring surrounding the hematoma. In the T2 FLAIR-weighted sequence, there was remarkable edema in the perihematomal region. At day 30, the edema remitted, and there was a clear area of red signal in the area surrounding the hematoma (Figure 1B).

The ipsilateral R2* value was 40.27 ± 11.62, 41.92 ± 13.56, and 60.89 ± 14.09 at day 1, 14, and 30, respectively (Figure 1C). The contralateral R2* value was 19.59 ± 2.34 at day 1, 21.16 ± 4.37 at day 14, and 19.76 ± 4.00 at day 30 (Figure 1C). The R2* value was significantly higher in the ipsilateral side than the contralateral side at day 1 (*t* = 7.17, *df* = 16, *p* < 0.01), day 14 (*t* = 6.92, *df* = 21, *p* < 0.01), and day 30 (*t* = 12.88, *df* = 14, *p* < 0.01). The R2* value at day 30 was significantly greater than day 1 (*t* = 4.14, *df* = 6, *p* < 0.01) and day 14 (*t* = 5.93, *df* = 11, *p* < 0.01) (Figure 1C).

At day 14, the R2* value decreased from 34.28 to 14.18 within 5 mm of distance. There was a logistic decay of R2* value in the perihematomal area from adjacent to the distal location (*R*^2^ = 0.99, *n* = 6, *p* < 0.01) (Figure 1E).

### The Relationship Between R2* Value Adjusted by Volume With Brain Edema, Brain Atrophy, Demographic Data, Vascular Risk Factors, and Clinical Characteristics

The brain edema volume was 17.69 ± 9.65 cm^3^, 26.25 ± 17.71 cm^3^, 9.76 ± 10.61 cm^3^ at day 1, day 14, and day 30, respectively. At day 1 and day 14, R2* value adjusted by volume was correlated with brain edema (*R*^2^ = 0.30, *n* = 15, *p* = 0.03 and *R*^2^ = 0.40, *n* = 21, *p* < 0.01, respectively) (Figure 2D and Supplementary Figure 1A). At day 30, the correlation between R2* value adjusted by volume and brain edema was not significant (*R*^2^ = 0.26, *n* = 14, *p* = 0.06) (Supplementary Figure 1C).

Of the 12 patients who had MRI at follow up, brain volume was 13.43 ± 12.49 cm^3^. The MMSE and MoCA scores at follow-up were 29.50 ± 0.80 and 27.33 ± 2.10, respectively. The R2* value was adjusted by volume at day 14 and correlated with brain atrophy at follow-up (*R*^2^ = 0.77, *n* = 9, *p* < 0.01) (Figure 2E). The correlation between R2* value was adjusted by volume at day1/day30 and brain atrophy was not significant (Supplementary Figures 1B,D).

There was a significant difference of age (*p* = 0.029) and initial hematoma volume (*p* < 0.01) between the high and low R2* value adjusted by volume groups (Table 2).

**Table 2:** Relationships of R2^*^ value adjusted by volume and clinical data (*N* = 21).

	Low R2^*^ value adjusted by volume group (*N* = 10)	High R2^*^ value adjusted by volume group (*N* = 11)	***P***
**Demographic data**			
Age, y	63.70 ± 8.06	54.27 ± 9.99	0.029^*^
Male, No. (%)	8 (80.00%)	10 (90.91%)	0.476
**Medical history, No. (%)**			
Hypertension	8 (80.00%)	7 (63.64%)	0.407
Diabetes	1 (10.00%)	4 (36.36%)	0.157
Smoking	7 (70.00%)	6 (54.55%)	0.466
**Admission clinical data**			
Glasgow Coma Scale score	15.00 (5.00)	15.00 (1.00)	0.863
NIHSS score	8.10 ± 5.07	6.18 ± 2.56	0.280
Systolic blood pressure, mm Hg	167.60 ± 32.03	158.45 ± 23.92	0.465
Diastolic blood pressure, mm Hg	96.90 ± 12.74	102.64 ± 16.39	0.385
**Imaging data**			
Lobar location of ICH, No. (%)	3 (30.00%)	1 (9.09%)	0.223
Hematoma volume on admission CT, cm^3^	10.19 ± 5.10	18.37 ± 7.44	0.009^**^
**Laboratory data**			
Leukocyte, 10^9^/L	6.58 ± 1.46	7.19 ± 2.07	0.450
Hemoglobin, g/L	142.50 (32.00)	150.00 (23.00)	0.557
Platelets, 10^12^/L	192.20 ± 48.78	190.91 ± 43.27	0.949
Glucose, mmol/L	5.44 (1.67)	6.37 (2.29)	0.099
TCHO, mmol/L	4.47 (1.42)	4.79 (1.01)	0.173
TG, mmol/L	1.00 (0.94)	1.61 (1.05)	0.061
LDL, mmol/L	2.43 ± 0.72	2.81 ± 0.67	0.218
HDL, mmol/L	1.20 (0.39)	1.01 (0.53)	0.223
Creatinine, μmol/L	90.01 ± 6.88	92.04 ± 18.54	0.749
PT, s	10.30 (0.88)	10.40 (1.40)	0.912
INR	1.02 (0.11)	1.00 (0.12)	0.573
Homocysteine, mmol/L	28.89 (39.65)	15.19 (10.45)	0.142
**3-month outcome**			
Modified rankin score	2.00 (0.75)	2.00 (1.00)	0.349

* *p < 0.05*,

** *p < 0.01*.

## Discussion/Conclusion

In this study, we aimed to validate the dynamic assessment of brain iron concentrations in perihematomal tissue after ICH with MR imaging. There were several major findings in the current study: (1) the iron concentration in the perihematomal region was progressively increased during the first month after ICH; and (2) the brain edema in the acute stage and brain atrophy in the later stage correlated with brain iron overload after ICH.

In the current study, the iron concentration in the perihematomal region began to increase within 24 hours after ICH. At day 1, the iron concentration was about 2 times more than the contralateral side. It continued to progress in the first month. At day 30, the iron concentration was approximately 3 times more than the contralateral side. In a rat model of ICH, the iron content reached its maximum at day 14 and was 2 times greater than the contralateral side (13). In comparison with the animal model, iron overload was more prominent and lasted longer than in human patients. Although the R2* value reached its maximum during the last scan, iron accumulation may still be ongoing afterward. Therefore, further investigations are needed to identify the peak time and time course of iron accumulation. When translating iron chelating therapy from an animal model to a human one, species difference should be considered, and the treatment course may be expanded to achieve a better effect.

There has been a continuous effort to explore a method for decreasing secondary injury and improving the prognosis of ICH. For example, deferoxamine has been shown to reduce brain injury by attenuating brain edema (9). The quantification of iron overload provides useful information in defining the therapeutic time window and the optimal duration of these treatments. Although quantitative susceptibility mapping (QSM) is more accurate for iron quantification (18), SWI is a widely available sequence and can be used in the routine clinical evaluation of iron overload after ICH.

In this study, we did not find a significant increase in ipsilateral R2* values from day 1 to day 14. Both the pseudocolor image and the R2* values showed that the evaluation of iron concentration was greatly influenced by the distinguished brain edema in ICH patients. When measured by serial MRI, perihematomal edema (PHE) was progressive and reached its maximum 14 days after onset. The development of PHE probably resulted in the plateau of R2* value from day 1 to day 14. Although the R2* value is strongly affected by iron concentration, the water content of brain tissue can also influence the R2* value.

The R2* value showed logistic decay in the perihematomal area. On the fourteenth day after ICH, the R2* value decreased from 34.28 to 14.18 in 5 mm of distance. This region manifested the migration of iron overload and the accumulation of hemosiderin in the perihematomal area, which represented the vulnerable tissue and secondary injury after ICH. The migration of iron can be visualized in ESWAN images from the hematoma to the perihematomal region. The highest iron concentration was located mainly inside the hematoma on day1. At this stage, iron was mostly stored in red blood cells, and hemoglobin as erythrocyte lysis begins. During this period, iron was mainly stored as ferritin and hemosiderin (19). Previous studies indicate that ferritin reached its maximum on days 7 to 14 after ICH (20, 21), while hemosiderin began to accumulate 6 days after ICH (22). Therefore, hemosiderin may be the major protein responsible for iron measurement at later stages.

In the current study, we defined R2* value adjusted by volume, a new indicator of perihematomal iron overload. In the previous FeCl_2_ infusion ICH experiment, simply iron overload resulted in acute brain edema and chronic brain tissue loss (23). However, there were few non-invasive methods to measure iron overload in ICH patients in the past, e.g., serum ferritin level. The current study indicated that R2* value adjusted by volume may be a marker of iron overload and a predictor of brain edema and atrophy. In this study, we used the volume difference between the two hemispheres as tissue loss, which can partially offset normal aging atrophy. In addition, the median atrophy rate of normal subjects was approximately −0.38% per year (24), which can be neglected compared to ICH injury.

Some limitations of the study must be noted. (1) The location of hematomas varies in our study. Although most of the hematomas were located in the basal ganglia, there were some patients with lobar hemorrhage. Iron accumulation in different brain structures is different as a consequence of normal aging. Additionally, R2* values are influenced by the myelin content in the brain (25). The heterogeneity of clinical characteristics should be considered when analyzing the results in this study. (2) Age-related differences in iron deposition in the brain are region specific. The basal ganglia were the location where the R2* value was greatly related to older age (26). In our cohort, the age of all patients ranged from 37 to 83 years old. Therefore, the iron deposition caused by aging cannot be excluded, although the dynamic change in iron overload was mainly due to the degradation of hemoglobin released from the hematoma. (3) Our study is also limited due to the small sample size.

In conclusion, the iron concentration in the perihematomal region progressively increased during the first month after ICH. R2* value adjusted by volume may be a predictor of brain edema and atrophy.

## Data Availability Statement

The original contributions presented in the study are included in the article/Supplementary Materials, further inquiries can be directed to the corresponding authors.

## Ethics Statement

The study was approved by the ethics committee of Peking University Health Science Center and Michigan University Joint Institute, and informed consent was obtained from the patients or their surrogates.

## Author Contributions

RL contributed to the acquisition of data, data analysis, and interpretation. HZ contributed to data analysis, interpretation, and manuscript preparation. SC contributed to the acquisition of data and revision of the manuscript. YS and HL contributed to the acquisition of data and data analysis. JX contributed to study concept and design, image analysis, and interpretation. YH contributed to study concept, design, and supervision. All authors contributed to the article and approved the submitted version.

## Conflict of Interest

The authors declare that the research was conducted in the absence of any commercial or financial relationships that could be construed as a potential conflict of interest.

## Supplementary Material

Supplementary Figure 1 **(A,C)** Correlation between R2* value adjusted by volume and brain edema at day 1 **(A)** and day 30 **(D)**. **(B,D)** Correlation between R2* value adjusted by volume at day 1 **(B)**/day 30 **(D)** and brain atrophy at follow-up.
