# Supplementary figures and images for: Association of Brain Iron Overload With Brain Edema and Brain Atrophy After Intracerebral Hemorrhage
Source: Front Neurol. 2020 Dec 18;11:602413. doi: 10.3389/fneur.2020.602413 (PMC7775517; doi:10.3389/fneur.2020.602413)

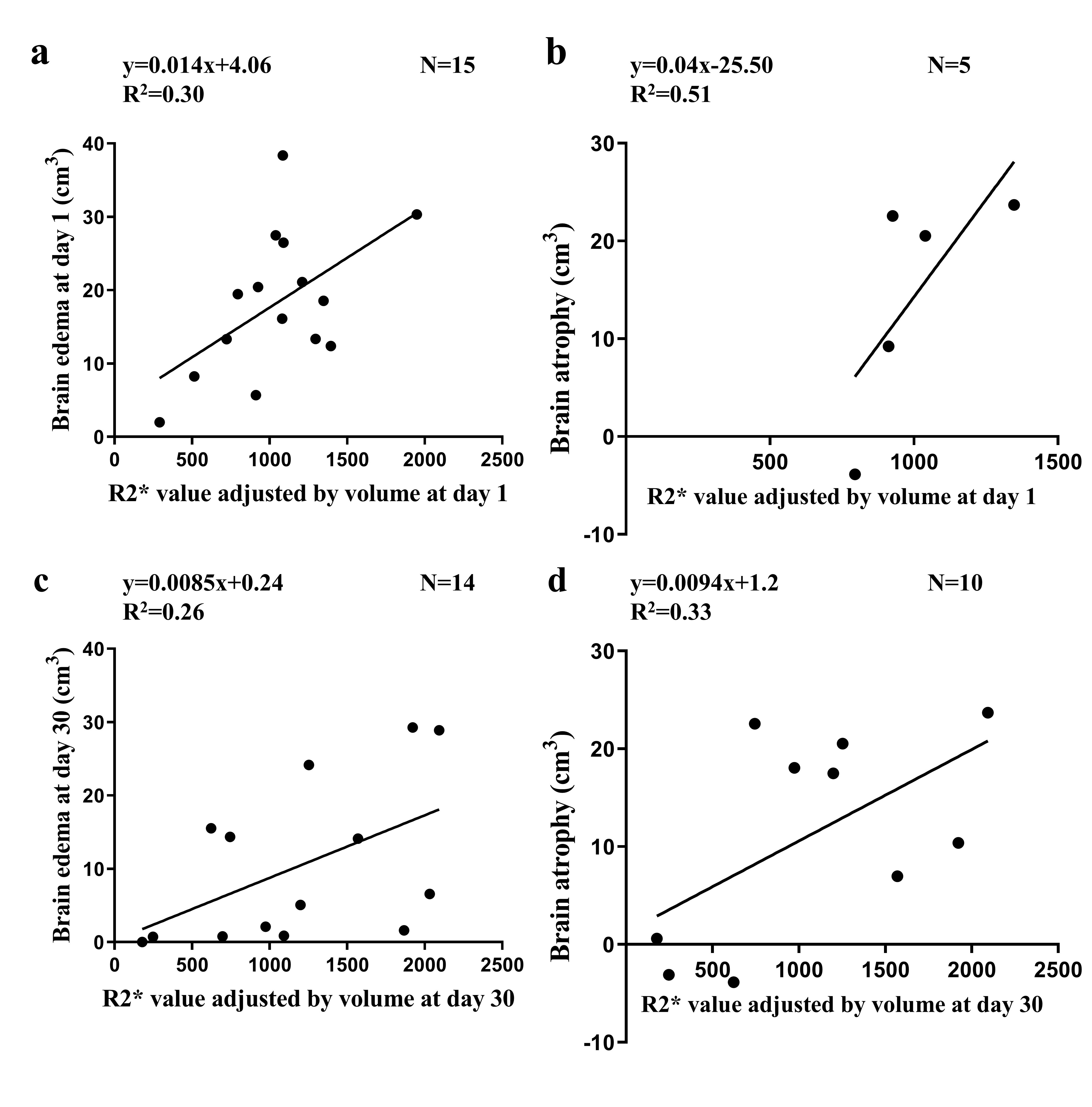

Supplement: Supplementary Figure 1 — (A,C) Correlation between R2* value adjusted by volume and brain edema at day 1 (A) and day 30 (D). (B,D) Correlation between R2* value adjusted by volume at day 1 (B)/day 30 (D) and brain atrophy at follow-up. [file Image_1.JPEG]
